# The association of ALT to HDL-C ratio with type 2 diabetes in 50–74 years old adults: a population-based study

**DOI:** 10.1038/s41598-024-60092-9

**Published:** 2024-04-24

**Authors:** Abolfazl Emamian, Mohammad Hassan Emamian, Hassan Hashemi, Akbar Fotouhi

**Affiliations:** 1 Student Research Committee, Shahroud University of Medical Sciences, Shahroud, Iran; 2 Ophthalmic Epidemiology Research Center, Shahroud University of Medical Sciences, Shahroud, Iran; 3 Noor Research Center for Ophthalmic Epidemiology, Noor Eye Hospital, Tehran, Iran; 4 Department of Epidemiology and Biostatistics, School of Public Health, Tehran University of Medical Sciences, Tehran, Iran

**Keywords:** ALT, HDL-C, Diabetes, Risk factors, Iran, Diseases, Endocrinology

## Abstract

There is limited information about the relationship between diabetes mellitus (DM) and ALT to HDL-C ratio. This study aims to investigate this relationship for the first time in Iran. The data of this study were taken from the third phase of the Shahroud Eye Cohort Study, which was conducted in 2019 with the participation of 4394 people aged 50–74. ALT and HDL-C levels were measured using a BT-1500 autoanalyzer. The mean ALT/HDL-C ratio was reported along with 95% confidence intervals (CI). The multiple logistic regression was used to examine the association between this ratio and DM, while controlling for the effects of other independent variables. The mean and standard deviation of the ALT/HDL-C ratio in all participants were 16.62 ± 11.22 (95% CI 16.28–16.96). The prevalence of DM was 34.7% and individuals with DM had a mean ALT/HDL-C ratio that was 1.80 units higher than those without diabetes (*P* < 0.001). Also, in individuals with DM, the HDL-C was found to be 0.035 (mmol/L) lower (*P* < 0.001), while ALT was 1.13 (IU/L) higher (*P* < 0.001) compared to those without diabetes. Additionally, after controlling for confounding factors, the odds of developing DM increased in a non-linear manner with an increase in the ALT/HDL-C ratio. Abdominal obesity, advanced age, female gender, and hypertension were also found to be associated with increased odds of DM. In conclusion, an increase in the ALT/ HDL-C ratiowas associated with higher odds of DM. This ratio can serve as an important predictor for diabetes mellitus.

## Introduction

Diabetes mellitus (DM) is a chronic disease characterized by high blood glucose levels. If not properly controlled, it can lead to many complications in organs such as the heart, eyes, and kidneys^1^. The prevalence of DM has been steadily increasing over the years. From 1980 to 2014 it rose from 4.3 to 9.0% in men and from 5.0 to 7.9% in women worldwide^2^. According to a report by the International Diabetes Federation in 2021, the global prevalence of DM among people aged 20–79 was 10.5%.It is projected to reach 12.2% by 2045^3^. Alarmingly, nearly half of people with DM remain undiagnosed^4^. According to the Global Burden of Disease reports in 2019, DM is the eighth cause of death and disability in the world and it is a major risk factor for ischemic heart disease and stroke which are the top two causes of death and disability^5^. Risk factors for DM can be categorized into six groups: environmental factors, smoking, alcohol consumption, high body mass index (BMI), low physical activity, and unhealthy diet. The most significant risk factor for developing is high BMI, followed by an unhealthy diet^6^. Other factors that increase the risk include a family history of DM, advancing age^7^, low levels of HDL cholesterol^8^, and hypertension^9^. Alanine transaminase (ALT) serves as a crucial marker for liver damage. Elevated levels of ALT are associated with an increased probability of DM^10–13^. Notably, this association follows a linear trend^10^. High ALT is the most common abnormality observed in liver function tests among individuals with DM^14^. Ratios of ALT to other biomarkers have also been investigated in the prediction of diabetes. For example, the ratio of ALT to AST has a positive relationship with the incidence of gestational diabetes^15^. Also, the ratio of AST to ALT has a non-linear and negative association with the risk of DM^16^, and can be used for prediction of diabetes^17^.

HDL-C is a lipoprotein which is known to prevent heart diseases. Although there is evidence that low HDL cholesterol or its high variability is an independent predictor of diabetes^18^, genetic studies do not confirm this relationship, suggesting that the relationship observed in descriptive studies may be confounded or caused by reverse causation^19^. The relationship between ALT/HDL-C ratio and DM is non-linear. Individuals with a higher ratio are more likely to develop DM^20^. Compared to other tests such as ALT, GGT, AST, TG, TC, waist circumference, HDL-C and even BMI, the ALT/HDL-C ratio demonstrates superior predictive ability for DM^20^. Recently, a longitudinal study with a follow-up period of 3.1 years revealed that the ratio of ALT to HDL was positively associated with the prediction of diabetes. This ratio was found to be superior to considering ALT and HDL-C separately^21^. The ALT to HDL-C ratio has been introduced as a combined marker for early diagnosis of DM. There have been very few studies investigating the association of this marker and DM. It is important to conduct large, community-based studies in various age groups to further investigate this association. At the best of our knowledge, there is no other study in this regard in Iran. This population-based study examines the association between ALT/HDL-C and type 2 diabetes mellitus for the first time in an Iranian community.

## Methods

This cross-sectional study was conducted using the data from the third phase of the Shahroud Eye Cohort Study (ShECS). This cohort study was conducted in three phases in Shahroud, northeast Iran. The first phase was conducted in 2009 with the participation of 5190 people aged 40–64. The details of sampling method of this study have been previously reported^22^. In short, 6311 people were randomly selected using the cluster sampling method in 300 clusters and among them 5190 people participated. The third phase of ShECS was conducted in 2019 on 4394 participants of the first phase. During this phase, in addition to comprehensive eye examinations and optometry, the following assessments were conducted: anthropometry, blood pressure measurement, blood glucose, HbA1c, lipid profile, ALT, AST, and CRP.

Individuals with HbA1c greater than or equal to 6.5% and/or fasting blood glucose greater than or equal to 126 (mg/dL) and/or treated with blood sugar lowering medications were defined as individuals with DM. The blood pressure of the patients was measured while sitting, from the right arm twice with an interval of 3 min by trained nurses. If the difference in systolic blood pressure was more than 10 mmHg or diastolic blood pressure was more than 5 mmHg, the blood pressure was measured for the third time. The average of two systolic and diastolic blood pressures that were closer to each other was regarded as final systolic or diastolic blood pressures^23^. People with systolic blood pressure greater than or equal to 140 mmHg or a diastolic blood pressure greater than or equal to 90 mmHg, and/or people treated with blood pressure lowering medication, were defined as having hypertension. All lab tests were performed using a BT-1500 autoanalyzer at a temperature of 37 °C. Weight was measured using a portable digital scale (Beurer PS-07, Germany) with an accuracy of one tenth kilogram and height was measured with an accuracy of 0.1 cm while standing and without shoes. Body mass index (BMI) was calculated by dividing weight (in kilograms) by height squared (in meters).

### Statistical analysis

Quantitative variables were reported as mean, standard deviation and 95% confidence intervals (CI). Number and percentage with 95% CI were reported for categorical variables. One-way ANOVA and t-test were performed to compare the mean of ALT/HDL-C, ALT, and HDL-C in groups of independent variables. Simple and multiple logistic regression was used to investigate the association between DM and ALT/HDL-C. The ALT to HDL-C ratio was dichotomized using the cutoff, provided by He et al.^21^. The association of this new variable with DM was assessed in another multiple logistic regression model. The effect of cluster sampling was considered in calculating standard error and confidence intervals.

### Ethical considerations

Shahroud Eye Cohort Study was approved by the ethics committee of Shahroud University of Medical Sciences (Reference numbers: IR.SHMU.REC. 1398.039). All methods were carried out in accordance with the Helsinki tenets and other relevant guidelines and regulations. Written informed consent was obtained from all participants.

## Results

**Figure 1:**
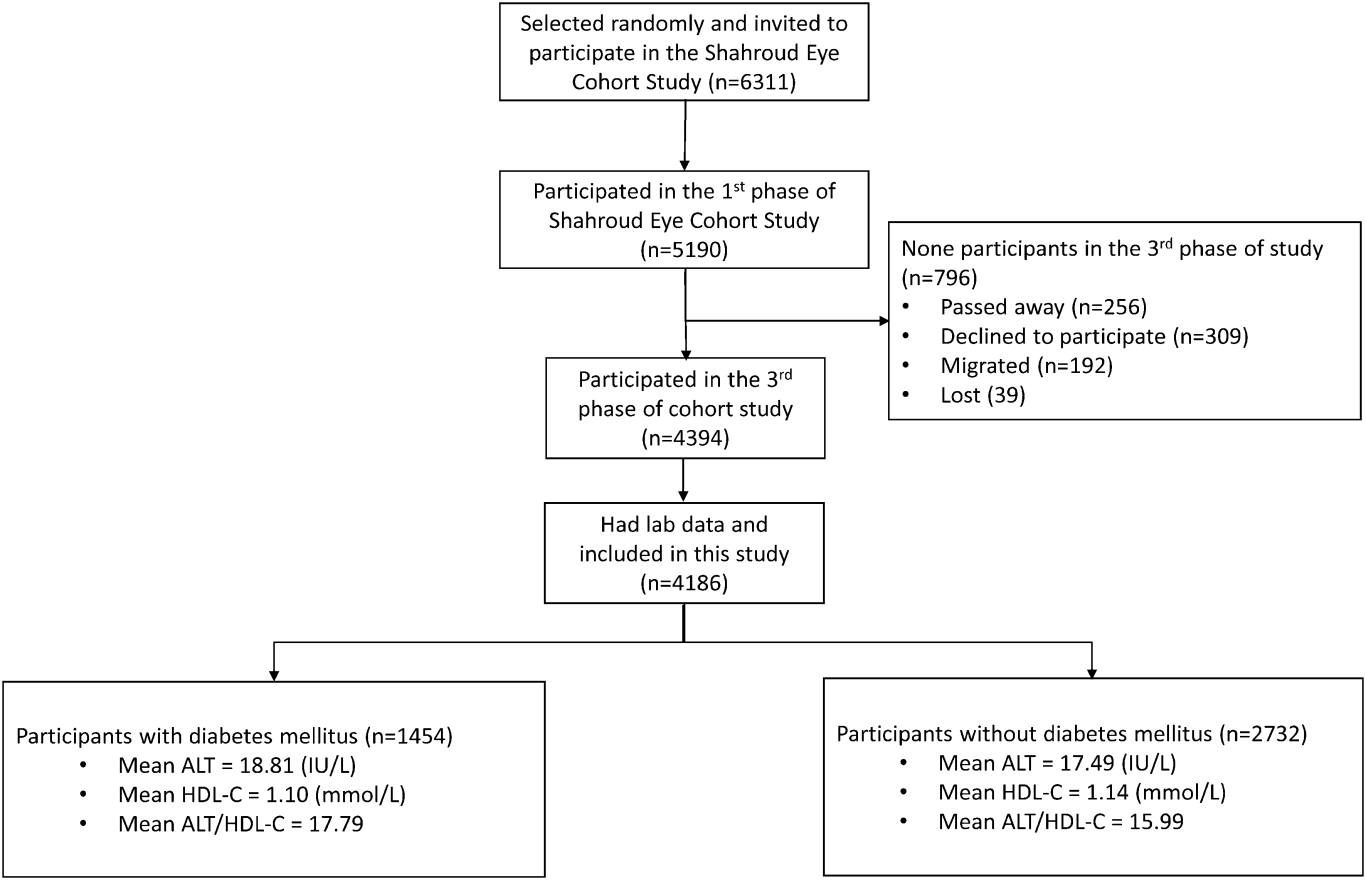
The flow diagram of study participants.

**Figure 2:**
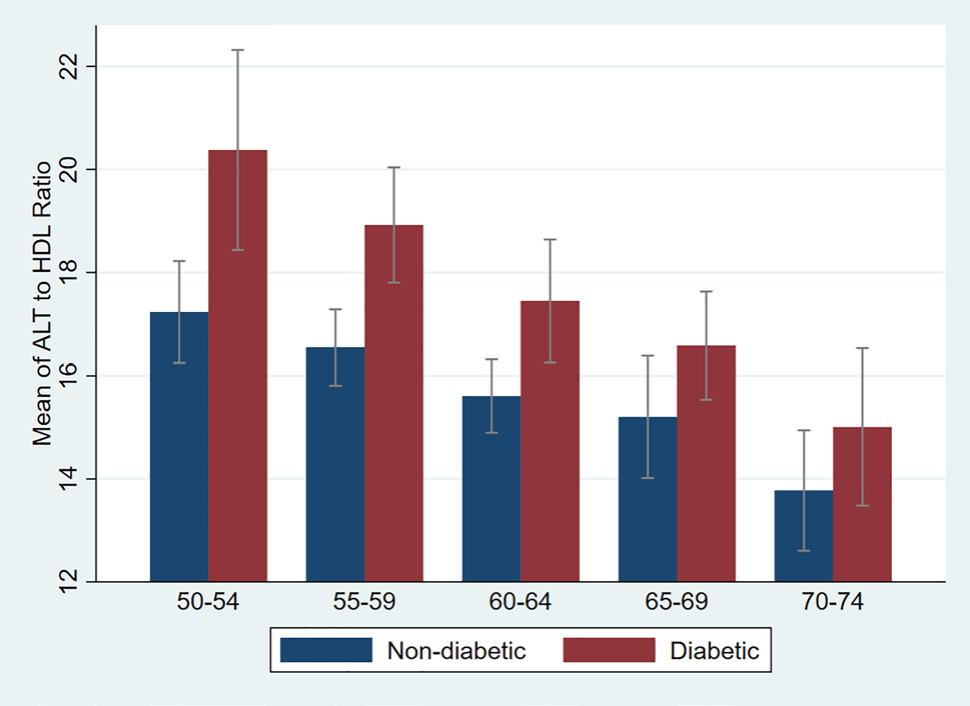
The mean and 95% confidence intervals of ALT to HDL-C ratio by age and diabetes groups.

Among the 4394 people who participated in the third phase of Shahroud Eye Cohort Study, 4186 were eligible in this study (Fig. 1). The age range of the participants was between 50 and 74 with an average (± SD) of 61.1 ± 6.1 years, and 59.5% of them were women. The mean levels of ALT and HDL-C were 17.95 ± 11.13 (IU/L), and 1.12 ± 0.20 (mmol/L) respectively. The mean ratio of ALT/HDL-C was 16.62 ± 11.22. In total, 34.7% of the studied participants had DM, and the mean ALT and HDL-C in them were 1.33 (IU/L) higher (*P* < 0.001) and 0.035 (mmol/L) lower (*P* < 0.001) than the group without DM. Also, Table 1 shows that the mean ALT/HDL-C in people with DM was 1.80 higher than in the group without DM (*P* < 0.001). This ratio decreased with increasing age (Fig. 2).

**Table 1:** The mean, standard deviation (SD) and range of ALT, HDL-C and ALT to HDL-C ratio by sex, age groups and the presence of diabetes mellitus in urban adults of Shahroud, Iran.

Independent variables	*n*	ALT (IU/L)			HDL-C (mmol/L)			ALT/HDL-C		
		Mean (SD)	Range	*P* value	Mean ± SD	Range	*P* value	Mean ± SD	Range	*P* value
Total	4186	17.95 (11.13)	2.1–187.6	–	1.12 (0.20)	0.58–2.57	–	16.62 (11.22)	1.93–190.91	–
Sex										
Male	1694	18.75 (11.01)	2.1–151.0	< 0.001	1.06 (0.18)	0.58–1.89	< 0.001	18.41 (11.64)	1.93–142.42	< 0.001
Female	2492	17.40 (11.18)	2.7–187.6		1.17 (0.20)	0.67–2.57		15.39 (10.75)	2.29–190.91	
Age groups (Year)										
50–54	798	19.47 (12.46)	4.3–151.0	< 0.001	1.13 (0.21)	0.67–1.78	0.210	18.08 (12.90)	3.33–149.40	< 0.001
55–59	1156	18.63 (11.08)	3.5–186.0		1.11 (0.19)	0.65–2.57		17.32 (10.75)	2.29–146.19	
60–64	1078	17.57 (10.10)	2.4–136.8		1.12 (0.20)	0.58–2.01		16.27 (10.52)	1.95–107.62	
65–69	727	17.14 (11.40)	2.1–187.6		1.13 (0.20)	0.64–1.89		15.74 (11.40)	1.93–190.91	
70–74	427	15.58 (10.03)	4.2–126.0		1.13 (0.21)	0.70–1.81		14.30 (9.86)	3.31–110.74	
Diabetes mellitus										
Yes	1454	18.81 (11.19)	3.5–151.0	< 0.001	1.10 (0.20)	0.63–1.89	< 0.001	17.79 (11.61)	2.29–142.42	< 0.001
No	2732	17.49 (11.07)	2.1–187.6		1.14 (0.20)	0.58–2.57		15.99 (10.96)	1.93–190.91

The results of multiple logistic regression in Table 2 shows that compared to people with ALT/HDL-C less than 10, the odds of DM was higher in groups with ALT/HDL-C above 10. The odds of DM in people with ALT/HDL-C of 50 to 59.9 was 2.36 (1.01–5.52) higher than reference group of less than 10 (*P* = 0.048). Also, the odds of DM was higher in people with hypertension, and in women. One centimeter increase in waist circumference was associated with a 4% increase in the odds of DM.

**Table 2:** The association of ALT/HDL-C with diabetes, adjusted with other covariates in multiple logistic regression model.

Independent variables	Simple model		Multiple model	
	OR (95% CI)	*P* value	OR (95% CI)	*P* value
ALT/HDL-C groups				
< 10	Reference group	–	Reference group	–
10–19	1.31 (1.11–1.55)	0.001	1.26 (1.07–1.50)	0.006
20–29	1.64 (1.33–2.03)	< 0.001	1.62 (1.30–2.03)	< 0.001
30–39	1.92 (1.38–2.68)	< 0.001	2.05 (1.42–2.96)	< 0.001
40–49	2.00 (1.15–3.48)	0.015	2.15 (1.18–3.92)	0.013
50–59	2.71 (1.22–6.01)	0.014	2.36 (1.01–5.52)	0.048
60–69	2.00 (0.78–5.12)	0.149	2.24 (0.82–6.13)	0.117
> = 70	1.87 (0.87–4.02)	0.106	2.27 (1.01–5.09)	0.046
BMI (Kg/m^2^)	1.05 (1.02–1.08)	< 0.001	NR	–
Female sex	1.58 (1.37–1.81)	< 0.001	1.67 (1.43–1.94)	< 0.001
Waist circumference (Cm)	1.05 (1.04–1.05)	< 0.001	1.04 (1.03–1.04)	< 0.001
Hypertension	2.30 (2.00–2.64)	< 0.001	1.80 (1.55–2.10)	< 0.001
Age groups (Year)				
50–54	Reference group	–	Reference group	–
55–59	1.32 (1.09–1.59)	0.004	1.36 (1.11–1.68)	0.003
60–64	1.54 (1.27–1.87)	< 0.001	1.58 (1.29–1.94)	< 0.001
65–69	1.75 (1.43–2.15)	< 0.001	1.75 (1.41–2.18)	< 0.001
70–74	2.00 (1.59–2.52)	< 0.001	1.97 (1.55–2.51)	< 0.001
ALT (IU/L)	1.01 (1.00–1.02)	0.002	NR	–
HDL-C (mmol)	0.41 (0.30–0.57)	< 0.001	NR	–

*OR* Odds ratio, *CI* Confidence intervals, *ALT* Alanine transaminase, *HDL-C* High density lipoprotein-cholesterol, *BMI* Body mass index, *NR* Not retained in multiple regression model due to collinearity.

**Figure 3:**
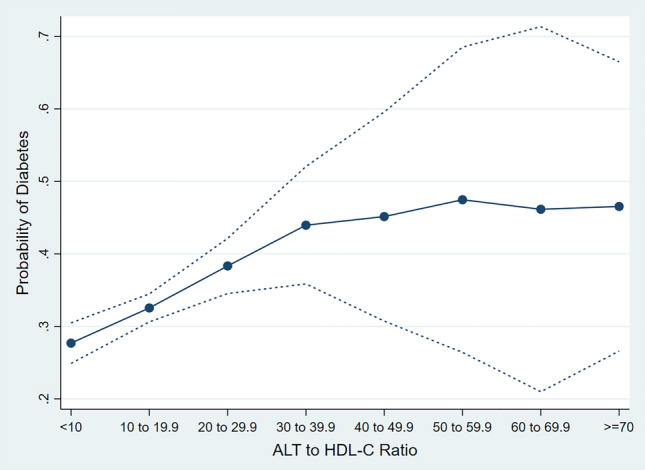
The association between ALT to HDL-C ratio and the probability of diabetes. The upper and lower lines represent 95% confidence intervals.

Table 3 presents the association between an ALT/HDL-C ratio greater than 14.9 (compared to less than 14.9) and DM, while adjusting for other covariates. Notably, the odds of DM increased by 44% when ALT/HDL-C levels exceeded 14.9. Finally, there was a non-linear relationship between the increase in ALT/HDL-C and the probability of DM, so that the increase in the ALT/HDL-C up to 60 increases the probability of DM, and at higher values, the probability of DM remains constant (Fig. 3).

**Table 3:** Association of ALT to HDL-C ratio > 14.9 with Diabetes Mellitus, Adjusted for other variables in multiple logistic regression model.

Independent variables	Odds ratio (95% CI)	*P* value
ALT/HDL-C groups		
≤ 14.9	Reference group	–
> 14.9	1.44 (1.27–1.64)	< 0.001
Female sex	1.64 (1.41–1.91)	< 0.001
Waist circumference (Cm)	1.04 (1.03–1.04)	< 0.001
Hypertension	1.81 (1.55–2.10)	< 0.001
Age groups (Year)		
50–54	Reference group	–
55–59	1.34 (1.10–1.65)	0.005
60–64	1.55 (1.26–1.90)	< 0.001
65–69	1.72 (1.38–2.14)	< 0.001
70–74	1.93 (1.51–2.45)	< 0.001

## Discussion

This cross-sectional and population-based study demonstrated a positive but non-linear association between the increase in ALT to HDL-C ratio and DM. This conclusion was obtained in a multiple regression model and after controlling of confounding variables such as waist circumference, hypertension and sex. To the best of our knowledge, this report is the first study in Iran that measured the association between this ratio and DM, furthermore, it is one of the few reports worldwide investigating this issue, with results similar to two other existing articles^20,21^. Our findings indicate that individuals with DM tend to have higher levels of ALT and lower levels of HDL-C. Other studies have also demonstrated that individuals with DM have higher ALT levels compared to those without DM, and that elevated ALT levels are directly linked to the development of DM^11–13,24–26^. Notably, there is a linear association between the probability of developing DM and ALT levels^10^. However, ALT does not possess any distinct advantage over other risk factors in predicting DM^11^. It has been reported that the HDL-C level in individuals with DM is lower compared to those without DM^26^. Also, HDL-C was negatively correlated with glycosylated hemoglobin^27^. The lower level of HDL-C, is associated with a higher risk of DM^18^. On the other hand, HDL-C has not been a reliable predictor for DM^28^. Therefore, when ALT increases and HDL-C decreases, the ALT/HDL-C ratio increases and can be a more effective predictor for DM compared to other risk factors^20^. It is important to note that over time, DM is associated with metabolic syndrome and other conditions such as hypertension and dyslipidemia, which are associated with reduced HDL-C levels. Insulin resistance and elevated fatty acid levels play an essential role in development of these conditions^29,30^.

The mechanism underlying the relationship between ALT/HDL-C and DM remains unknown, but it could be attributed that high ALT has a strong relationship with insulin resistance^31,32^. Furthermore, increased fasting glucagon has been found to be associated with elevated ALT^31^. Additionally, there is a reverse association between ALT and Adiponectin^33^. Previous studies have indicated that low level of Adiponectin increases insulin resistance^34,35^. On the other hand, Adiponectin prevents the apoptosis of pancreatic beta cells and increases their lifespan^36^. Elevated ALT is considered one of the markers for non-alcoholic fatty liver disease (NAFLD), which is more prevalent in people with type 2 DM^37^. Also, high levels of HDL-C modulate glucose metabolism^38^, prevent apoptosis of pancreatic beta cells^39,40^, and increase glucose absorption in skeletal muscle cells^38^, which are proposed mechanisms for the association between HDL-C and DM.

As a secondary result, this study showed that hypertension is one of the risk factors of DM. A previous article of ShECS has also shown the association between hypertension and DM^41^. Other studies, have identified hypertension as a risk factor for DM as well^9,42^.

The results of this study showed that the prevalence of DM in women was higher compared to men, which aligns with the findings of the previous phase of the ShECS. This difference is likely due to the higher prevalence of obesity among women in this age group^43^.

After controlling for other risk factors, our findings demonstrated a robust and noteworthy association between waist circumference and DM. This outcome aligns with previous studies that have identified abdominal obesity, regardless of BMI, as a risk factor for heart diseases, DM, and high blood pressure^44,45^. Waist circumference is also a better predictor than BMI for comorbidities^45^. Additional research has indicated that people with abdominal obesity or central obesity exhibit greater severity of insulin resistance and a higher likelihood of developing DM compared to those without these risk factors. Independently, abdominal obesity exhibits a significant association with the risk of DM^46,47^.

High sample size, population-based sampling, conducting examinations and tests with good quality control and assurance, as well as using appropriate analysis methods are the strengths of this study. However, there are also some limitations to consider. Firstly, it is important to note that the results of this study are based on the third phase of the ShECS, meaning that causal inferences cannot be made due to the cross-sectional nature of the study. Secondly, it is worth mentioning that certain lab tests such as GTT, were not measured in this study. Lastly, the age group of the studied population was limited to individuals between 50 and 74 years old. The results of this study can be utilized to construct a diabetes prediction model. However, it is important to note that the development of a prediction model requires longitudinal studies, which was not a primary objective of this cross-sectional study.

Shahroud is a typical city in Iran, and like the entire country, the majority of its society is made up of the Fars race. No particular ethnicity dominates in the city. Additionally, the average literacy, age, gender composition, and most health indicators of the population are almost similar to the national average. Thus, the results of this study can be cautiously generalized to Iranians in the studied age groups.

## Conclusion

ALT to HDL-C ratio has a direct nonlinear association with DM, and it can be used as a combined marker to predict DM. More studies with cohort design are needed to confirm this issue. Increasing age, abdominal obesity, high blood pressure and female sex were associated with an increased odd of DM.

## Data Availability

Data are available from corresponding author (emamian@shmu.ac.ir) on reasonable request.
